# Investigation of KIR/HLA relationship and other clinical variables after T-cell-replete haploidentical bone marrow transplantation in patients with acute myeloid leukemia (AML)

**DOI:** 10.1186/s12865-023-00548-1

**Published:** 2023-06-20

**Authors:** Tahereh Bakhtiari, Mohammad Ahmadvand, Marjan Yaghmaie, Alireza Sadeghi, Seied Asadollah Mousavi, Tahereh Rostami, Mazdak Ganjalikhani-Hakemi

**Affiliations:** 1 Department of Immunology, School of Medicine, Isfahan University of Medical Sciences, Isfahan, Iran; 2 Cell Therapy and Hematopoietic Stem Cell Transplantation Research Center, Research Institute for Oncology, Hematology and Cell Therapy, Tehran University of Medical Sciences, Tehran, Iran; 3 Hematology, Oncology and Stem Cell Transplantation Research Center, Research Institute for Oncology, Hematology and Cell Therapy, Tehran University of Medical Sciences, Tehran, Iran; 4 Department of Internal Medicine, Faculty of Medicine, Isfahan University of Medical Sciences, Isfahan, Iran; 5 Hematologic Malignancies Research Center, Research Institute for Oncology, Hematology and Cell Therapy, Tehran University of Medical Sciences, Tehran, Iran; 6 Department of Immunology, Faculty of Medicine, Yeditepe University, Istanbul, Turkey

**Keywords:** KIR/HLA mismatch, KIR/HLA match, Acute myeloid leukemia, Post-transplant cyclophosphamide, Haploidentical Transplant, HSCT

## Abstract

**Background:**

KIR/HLA mismatch in hematopoietic stem cell transplantation (HSCT), particularly in patients with acute myeloid leukemia (AML), was related to decreased recurrence rates, improved engraftment, and a reduction in graft-versus-host disease, according to recent research (GVHD). Uncertainty exists about the impact of KIR/HLA mismatch on haploidentical-HSCTs treated with post-transplant cyclophosphamide (PTCy). We attempted to analyze the effects of KIR/HLA mismatch on clinical outcomes on transplant outcomes using the cohort of 54 AML patients who received a haplo-HSCT with PTCy.

**Results:**

In contrast to KIR/HLA match, our findings showed that donor KIR/HLA mismatch was substantially associated with superior OS (HR, 2.92; (*P* = 0.04)). Moreover, donor KIR/HLA mismatch (KIR2DS1_D_/C2^+^_R_ and KIR2DS2_D_/C1^+^_R_ mismatch versus KIR2DL1_D_/C2^−^_R_ mm, KIR2DL2/3_D_/C1^−^_R_ mm and KIR3DL1_D_/Bw4^−^ mm) was correlated with the improvements in OS (HR, 0.74; *P* = 0.085) and activating. KIR/HLA mismatch versus KIR/HLA match was significantly correlated with improvements in OS (HR, .46; *P* = 0.03) and inhibitory. KIR/HLA mismatch versus KIR/HLA match was enhancement in the OS (HR, .93; *P* = 0.06). Despite a higher rate of aGvHD (grade I-IV) in the patients with KIR/HLA mismatch compared to KIR/HLA matched (57% vs. 33% (*p* = 0.04). However, the KIR/HLA mismatch group saw a decreased relapse rate (3.2% vs. 23%, *p* = 0.04).

**Conclusion:**

This analysis shows the significance of KIR/HLA Incompatibility, other clinical variables like CMV, the relationship between donor/recipient and donor age, and the relationship between donor/recipient and donor age in the haplo-donor selection process. It also suggests that KIR and HLA mismatching between donor and recipient could be routinely performed for haplo-donor selection and may improve clinical outcomes after haplo-HSCTs with PTCy.

## Background

Despite the high chance of cure for acute myeloid leukemia (AML) following allogeneic hematopoietic stem cell transplantation (allo-HSCT), transplant-related mortality (TRM) in the absence of an HLA-matched sister donor (MSD) is a major downside of this method [1]. The easily available haploidentical donors, such as parents, kids, and nonidentical siblings, make HLA-haploidentical HSCT (haplo-HSCT), an attractive alternative technique for patients lacking an HLA-matched donor, conceivable [2]. Haplo-HSCT outcomes for AML patients have been successfully improved by recent developments in HSC transplantation techniques, including donor selection, conditioning regimen modification, graft-versus-host disease (GVHD) prophylaxis with post-transplant cyclophosphamide (PTCy) or ex vivo T cell-depletion, as well as supportive care [3, 4]. Relapse, however, continues to be the leading cause of post-transplant mortality despite tremendous advancements over the years. Leukemic cells escaping the immune system seem to be the primary cause. Downregulation of HLA class II molecules has been investigated as a mechanism of immune evasion [5]. Alloreactive NK cells have a strong graft-versus-leukemia (GvL) action that aids in the elimination of tumors [6, 7]. Natural killer (NK) cells, also known as granular lymphocytes, are a subset of innate immune cells that share a common progenitor with T and B cells in the bone marrow. They are typically recognized by the absence of the surface TCR and related CD3 molecules and the expression of the neural cell adhesion molecule (NCAM; also known as CD56) as CD3CD56 + cells, and they make up between 5–20% of all peripheral blood mononuclear [8–10]. NK cells that have cytolytic action against tumor cells and virus-infected cells release proinflammatory cytokines (GM-CSF, IFN, TNF, CCL1, CCL2, CCL3, CCL4, CCL5, and CXCL8) including interferon (IFN), tumor necrosis factor (TNF), and chemokines (CCL1, CCL2, CCL3, CCL4, CCL5, and CXCL8). These cytokines may also alter the activity of innate and adaptive immune cells [9, 11]. According to the "missing self" concept, NK cells may differentiate between normal and pathological cells through killer-cell immunoglobulin-like receptors, a set of activating and inhibiting receptors (KIRs). Major histocompatibility complex (MHC; also known as human leukocyte antigen [HLA] class I molecules)-deficient or -absent cells are one of the main tasks of NK cells [12, 13]. Innate effectors, namely NK cells, generate many inhibitory receptors that recognize HLA class I molecules. These inhibitory receptors bind to inhibitory KIRs, restrict NK cell activity, and prevent healthy self-cell death. Acting as antigen-presenting molecules for T cell or NK cell target receptors, they play a vital role in the adaptive immune response [14–16]. The human KIR gene family has been subdivided into four groups on chromosome 19q13.4 (KIR2DL1-5, KIR3DL1-3, KIR2DS1-5, and KIR3DS1). Six genes are produced by the activating KIR genes, which create activating receptors, as opposed to eight genes that are inhibitory KIR genes that encode inhibitory receptors [17–19]. Furthermore, two main KIR haplotype groups, the A and B haplotypes, have been discovered [20]. The KIR2DS4 activating gene is present in the A haplotype that contains the KIR3DL3, KIR2DL3, KIR2DL1, KIR2DL4, KIR3DL1, KIR2DS4, and KIR3DL2, too, while the B haplotype, that contains the KIR2DS1, KIR2DS2, KIR2DS3, KIR2DS5, and KIR3DS1 has up to five activating KIR genes [21–24]. Each individual may be classified according to one of two KIR genotypes: A/A or B/B, which are homozygous for the haplotypes of Groups A and B, respectively, and B/x, which is heterozygous for A and B (A/B). According to theory, B/x individuals possess more activating receptors, which may enhance their response to malignant or virus-infected cells [25]. The very diverse and polymorphic KIRs, which include the HLA-A, HLA-B, and HLA-C groups as classical and the HLA-E and HLA-G groups as nonclassical KIR ligands, accurately recognize epitopes shared by groups of either classical or nonclassical MHC class I alleles. KIR2DL1 recognizes HLA-C group 2 (C2) alleles, KIR2DL2 recognizes HLA-C group 1 (C1) alleles, and KIR3DL1 recognizes HLA-Bw4 alleles [26]. By secreting proinflammatory cytokines, HLA-C, on the other hand, is the main class I isotype involved in the inhibitory and activating modulation of NK cells to either protect against or trigger the lysis of tumor cells and virus-infected cells [27]. Thus, the mismatch between KIR and HLA molecules may have an effect on graft-versus-leukemia (GvL), which would also reduce the recurrence rate following HSCT [28]. These clinical studies and KIR immunogenetics research suggest that KIR-driven alloreactivity may be predicted more correctly if the donor KIR genotype is taken into consideration in addition to the recipient's HLA genotype [29]. According to the lack or presence of KIR/HLA mismatch, we examined the outcomes of AML patients who had T-cell-replete haploidentical hematopoietic stem cell transplantation in this single-center research.

## Results

### Patients and donors Characteristics

The study comprised 54 AML patients who had a haploidentical transplant utilizing PTCy. With a range of 8 to 43, the average patient age at transplantation was 18, and 78% of them were male. At the time of transplantation, 33 patients (62%) were in first remission (CR1), and 21 patients (38%) were in a second or later remission (CR >  = 2). Hematopoietic cell transplantation–specific comorbidity index was ≥ 3 in 38%. DRI was low, intermediate, and high/very high in 9%, 32%, and 13%, respectively. Patient features are presented in Table 1. The average age of the donors was 26. The donor was either a parent, a sibling, or a sibling who was 16 years of age or younger in 29.6%, 40.7%, and 29.6% of the instances, respectively. 20% of the transplants had both male and female donors and recipients. Pre-HSCT CMV serology revealed that 53.7% of the patients had a high risk CMV D-/R + status for CMV reactivation after transplantation. The features of the donor and transplant characteristics are listed in Table 1.

**Table 1:** Baseline information and clinical characteristics of patients and donors

Patients’ characteristics		
Age [Median range] (year)		18 [8–43]
Gender	Male Female	42 (78%) 12 (22%)
ABO compatibility	Compatible Minor inc Major inc	35 (64.8%) 12 (22.3%) 7 (12.9%)
HCT-CI	0–2 ≥ 3	33 (62%) 21 (38%)
Disease risk index (DRI)	Low Intermediate High/very High	9 (16.6%) 32 (59.2%) 13 (24.2%)
CMV	Recipient positive Recipient negative	34 (63%) 20 (37%)
Graft composition Median	Median CD34 + cells: 10*6 /kg Median CD3 + T cells: 10*7 /kg	6.5 (4.1–10.8) 22.3 (7.3–50.6)
Status at treatment	CR1 CR2	33 (62%) 21 (38%)
**Donor’s and transplant characteristics**		
Age [Median range] (year)		26 [7–61]
Gender match	FDMR Other	11 (20%) 43 (80%)
CMV serostatus (D/R)	+ / + − / + + / − − / −	4 (7.4%) 29 (53.7%) 4 (7.4%) 17 (31.5%)
Donor relationship	sibling ≥ 16 sibling ≤ 16 Parent	16 (29.6%) 22 (40.7%) 16 (29.6%)
HLA × /10 mismatch (GVH)	≤ 3 4 5	12 (22/2%) 14 (26%) 28 (51/8%)
Donor KIR haplotype	AA AB BB	17 (31.4%) 31 (57.4%) 6 (11.2%)
KIR/HLA mismatch	0 ≥ 1	24 (44.4%) 30 (55.6%)

*Inc* incompatible, *HCT-CI* Hematopoietic cell transplant comorbidity index, *CMV* Cytomegalovirus, *CR* Complete Remission, *FDMR* female donor–male recipient, *D* donor, *R* recipient

### Donor KIR and recipient HLA relationship

Thirty (55.6%) of the 54 donors with KIR genotyping had KIR/HLA mismatches. Table 2 displays the distribution of all donors' KIR genotypes (activating and inhibitory), as well as the presence or absence of their ligands.

**Table 2:** Characteristics of Inhibitory and Activatory KIR/HLA mismatches

Characteristics	**NO (%)**
HLA-C2 absent for donor KIR2DL1	11 (36.6%)
HLA-C1 absent for donor KIR2DL2,3	1 (3.3%)
HLA-Bw4 absent for donor KIR3DL1	5 (16.6%)
HLA-C1 absent for donor KIR2DL3	5 (16.6%)
HLA-C2 present for donor KIR2DS1	4 (13.3%)
HLA-C1 present for donor KIR2DS2	4 (13.3%)

For the donor NK cell benefit model, the NK cell alloreactivity was predicted based on high-resolution HLA typing of the donor and recipient. Briefly, KIR ligand HLA-C, HLA-B and HLA-A molecules were grouped into three major categories (C1, C2, Bw4) based on the specific amino acid sequence that defines specific KIR ligand binding https://www.ebi.ac.uk/ipd/kir/ligand.html.

### Overall survival (OS)

**Fig. 1:**
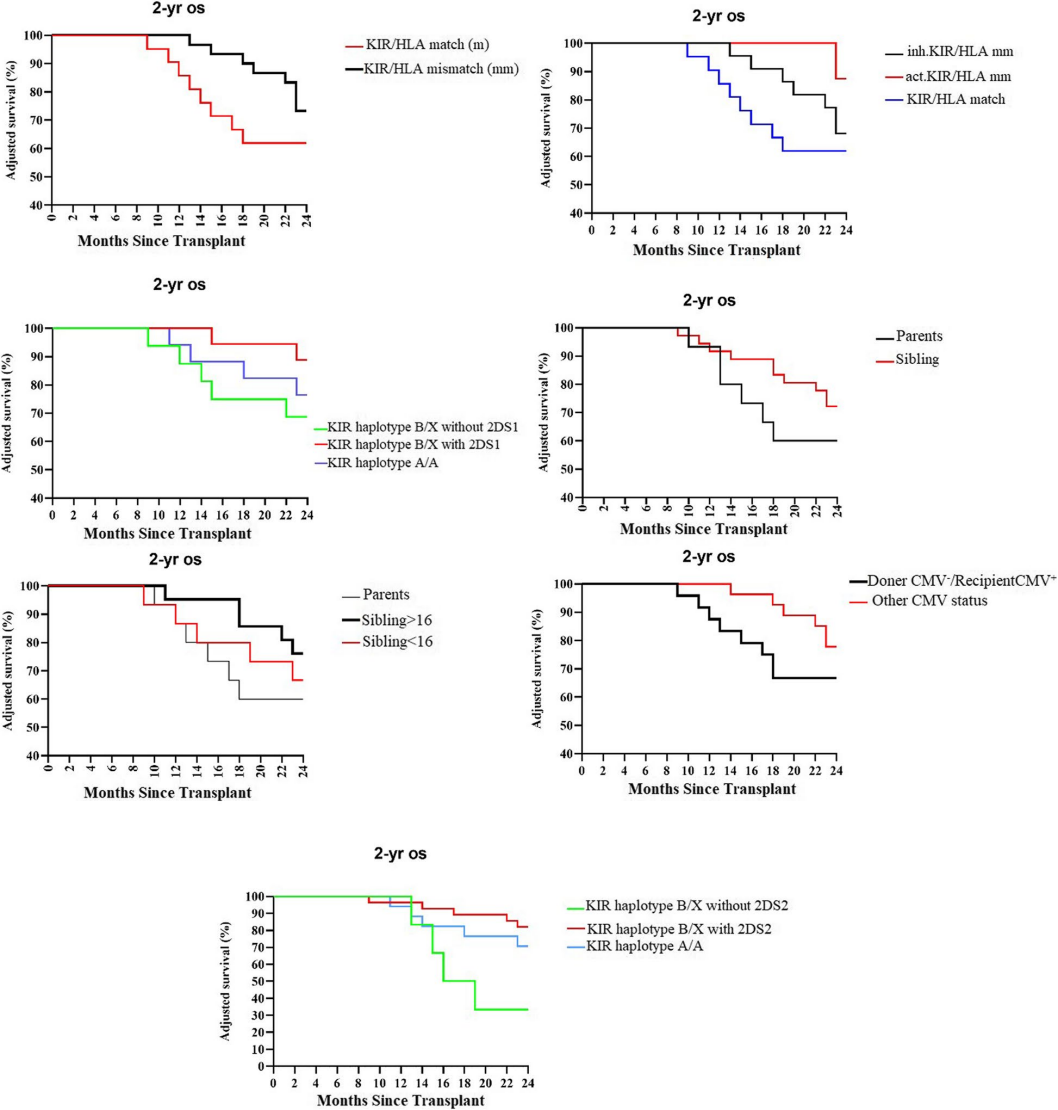
Adjusted Overall survival

To investigate the impact of the donor KIR genotype on patient outcomes, several NK alloreactivity models were utilized. Our findings (Table 3) revealed that donor KIR/HLA mismatch was substantially associated with superior OS (HR = 2.92; P 0.04) compared to KIR/HLA match (median follow-up, 18 months). The improvement in OS was also substantially correlated with activating KIR/HLA mismatch against KIR/HLA match (HR = 0.46; *P* = 0.03) and inhibitory KIR/HLA mismatch versus KIR/HLA match (HR = 0.93; *P* = 0.06). These inhibitory KIR2DL1/C and activating donor KIR/HLA mismatches of KIR2DS1/C2 and KIR2DS2/C1 Our results demonstrated that donation from a donor carrying KIR B/x with 2DS2 was related to a better OS (HR = 0.34; *P* = 0.05) compared to those with haplotype A/A. OS increased in patients who received transplants from KIR B/x haplotype donors who lacked KIR2DS2 (HR = 0.42; *P* = 0.006). Additionally, transplantation from a donor who had the KIR B/x haplotype and KIR2DS1 was associated with a better OS in comparison to donors who have the KIR A/A haplotype (HR = 0.63; *P* = 0.05) and KIR B/x haplotype but lacked KIR2DS1 (HR = 0.42; *P* = 0.001). The following Donor KIR and recipient HLA relationship feature improved 2-year adjusted OS: KIR B/x haplotype with 2DS2 (88%) compared to KIR A/A haplotype (79.1%) and KIR B/x haplotype lacking 2DS2 (57.3%), KIR B/x haplotype with 2DS1 (94%) compared to KIR A/A haplotype (79.1%) and KIR B/x haplotype without 2DS1 (76.1%) and KIR/HLA mismatch versus KIR/HLA match (87.5% vs. 61.9%) (Fig. 1).

**Table 3:** Two years OS based on donor KIR and recipient HLA relationship

Comparison groups	Patient number	Median OS (%)	HR	*P value*
KIR/HLA mismatch vs. KIR/HLA match	30 vs. 24	87.5 vs. 61.9	2.92	< 0.04
KIR B/x haplotype with 2DS2 vs. KIR A/A haplotype	20 vs. 17	88 vs. 79.1	0.34	0 .05
KIR B/x haplotype w/o 2DS2 vs. KIR A/A haplotype	17 vs. 17	57.3 vs. 79.1	0.63	0.01
KIR B/x haplotype with 2DS2 vs. KIR B/x w/o 2DS2	20 vs. 17	88 vs. 57.3	0.42	0.006
KIR B/x haplotype with 2DS1 vs. KIR A/A haplotype	24 vs. 17	94 vs. 79.1	0.63	0.05
KIR B/x haplotype w/o 2DS1 vs. KIR A/A haplotype	13 vs. 17	76.1 vs. 79.1	1.63	ns
KIR B/x haplotype with 2DS1 vs. KIR B/x w/o 2DS1	24 vs. 13	94 vs. 76.1	0.42	0.001
Activating KIR/HLA mismatch vs. KIR/HLA match	8 vs 24	94 vs 61.9	0.46	0.03
Inhibitory KIR/HLA mismatch vs. KIR/HLA match	22 vs 24	81 vs 61.9	0.93	0.06

w/o; without, ns; not significant

The donor-recipient connection and also donor age had considerable influence on post-transplant survival. Patients who got transplant from sibling > 16 years old had substantially superior survival compared to the patients transplanted from their parents (83% vs. 67.8% HR = 0.751 *P* = 0. 03). Although OS was greater in the patients who got transplant from their sibling < 16 years old (78.1% vs. 67.8%), it was not substantially different (HR = 1.75 *P* = NS). Finally, our results showed that CMV D^−^/R^+^ status compared to another donor and recipient CMV serostatus was related to a poorer OS (87/8% vs. 77% HR = 2.42 *P* = 0. 06).

Relapse Incidence.

**Fig. 2:**
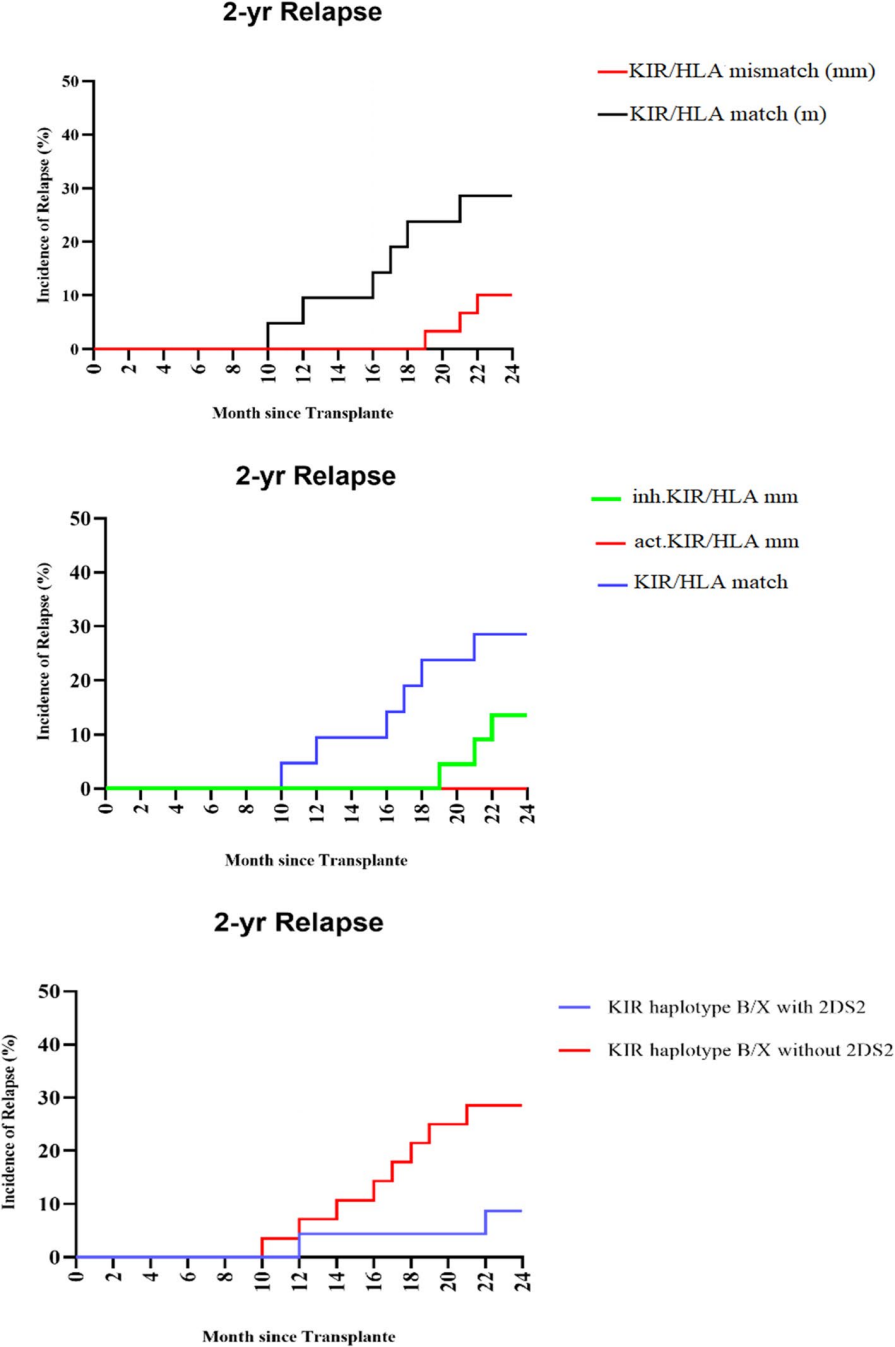
Adjusted incidence curves of relapse or progression according to donor characteristics

In comparison to KIR/HLA match patients, the KIR/HLA mismatch group saw a decreased recurrence rate (3.2% vs. 23.3%, *p* = 0.04). Patients who received organ transplants from donors by triggering KIR/HLA mismatches had a considerably reduced recurrence rate than those who did not (0% vs. 23.3%; *P* = 0.01) When compared to the lack of a mismatch, that was equivalent to an inhibitory KIR/HLA mismatch (2.8% vs. 23.3%, *P* = 0.04). Regarding KIR B/x haplotype 2DS1, although the relapse rate was higher in the absence of 2DS1 than from donors with 2DS1 (26.4% vs. 8.5%; *P* = 0.053), there was no statistically significant difference between donors with 2DS1 and donors without 2DS1 in the patients who received HSCT from donors with KIR B/x haplotype. Notably, there was no discernible difference in relapse rate between the KIR B/x and KIR A/A haplotypes. When compared to other CMV serostatus, CMV donor negative-recipient positive serostatus (D-/R +) was linked to a higher probability of recurrence (17.4% vs. 13.1%; *P* = 0.08). The corrected incidence curves of relapse or progression are shown in Fig. 2. The donor-recipient relationship was correlated with a lower risk of relapse; 9.1% of the patients transplanted from their siblings versus 13.2% who received HSCT from their parents (HR = 0 0.36; *P* = 0.45).

### Acute and Chronic GvHD

Patients with KIR/HLA mismatches had a higher probability of developing acute GvHD (57% vs. 33%; *P* = 0.04) (grade I-II: 27%; grade III-IV: 30%) compared to those with KIR/HLA matches (grade I-II: 12%; grade III-IV: 21%). Chronic GvHD was more common in individuals with KIR/HLA mismatches (27%), compared to 17% in patients with KIR/HLA matches (*p* = 0.06). Notably, neither the donor KIR A/A nor KIR B/x haplotypes nor the frequency of GvHD was correlated. Table 4 displays the prevalence of aGVHD and cGVHD.

**Table 4:** prevalence of aGVHD and cGVHD in AML patients

aGVHD			cGVHD	
	Patient a-GVHD ^+^ (Number, %)	Patient a-GVHD^−^ (Number, %)	Patient cGVHD^+^ (Number, %)	Patient cGVHD^−^ (Number, %)
KIR/HLA m (number, %)	8 (33%)	16 (67%)	4 (17%)	20 (83%)
KIR/HLA mm	17 (57%)	13 (43%)	8 (27%)	22 (73%)
Total	25 (46%)	29 (54%)	12 (22%)	42 (78%)

*aGVHD* acute graft versus host disease, *cGVHD* chronic graft versus host disease, *m* match, *mm* mismatch

### NRM

**Fig. 3:**
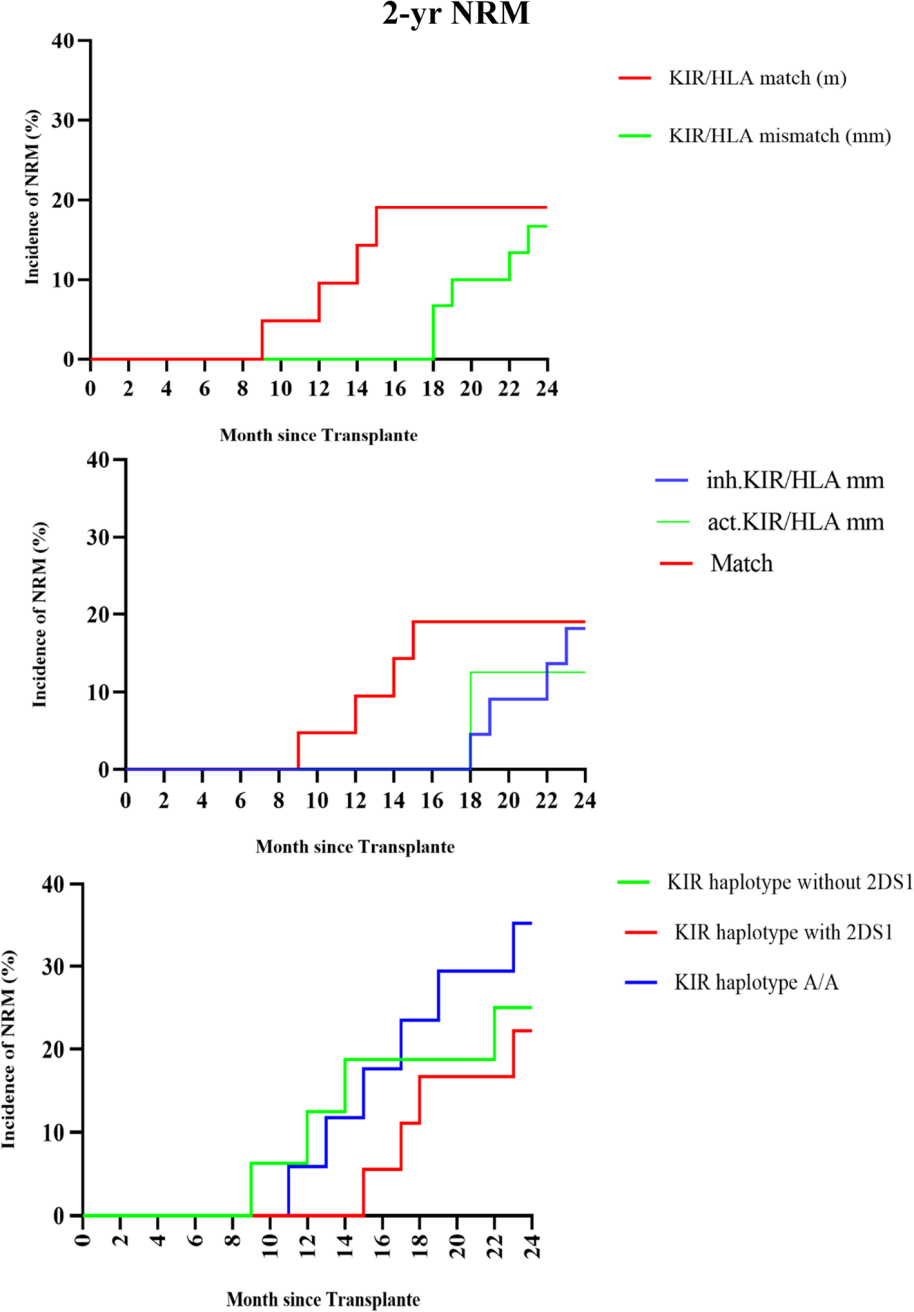
Adjusted incidence of NRM according to donor characteristic

Patients transplanted from donors with KIR A/A haplotype showed a higher incidence of NRM when compared to donors with KIR B/x haplotypes with 2DS1 (23% vs. 11% *P* = 0.047) and donors with KIR B/x haplotypes without 2DS1 (18% vs 11% *P* = NS) (median follow-up; 18 months). Patients with KIR-HLA mm had lower NRM rates. Patients who received HSCT from donors who had inhibitory KIR genes (KIR2DL1 and KIR2DL2/3 and KIR3DL1)/HLA-C mismatch and activating KIR genes-HLA-C mismatch experienced a lower incidence of NRM than patients who received HSCT from donors who had KIR/HLA matches, but this difference was not statistically significant (7.3% vs. 17.4% HR = 0.77 *P* = 0.14). Focusing on activating and inhibitory KIR/HLA mismatches, despite Inhibitory KIR/HLA mismatches resulted in a lower incidence of NRM compared to KIR/HLA matches (4.8% vs. 17.4% HR = 1.76 *P* = 0.063), it was not significant for activating KIR/HLA mismatches compared to KIR/HLA matches (11.3% vs 17.4% HR = 1.29 *P* = 0.6). Considering the relationship between recipient and donor, siblings > 16 were significantly associated with reduced risk of NRM (11% vs. 20.1% *P* = 0.07). About CMV serostatus and NRM, high-risk patients (D-/R +) had a higher incidence of NRM compared to other CMV serostatus (24% vs. 8.4%) (HR = 1.87 *P* = 0.051). Figure 3 shows the adjusted incidence of NRM curves.

#### Effect of recipient, donor, and HSCT-related characteristics on clinical outcomes

Results of univariate Cox regression analyses of the effect of baseline clinical characteristics on HSCT outcomes are shown in Table 5. We identified that higher HCT-CI (HR, 1.26; 95% CI, 1.16–1.34; *P* = 0.02) and high/very high DRI (HR, 1.86; 95% CI, 1.38–2.49; *P* = 0.04) were clinical factors associated with poor OS. Moreover, the result of the univariate analysis revealed that the effect of baseline clinical characteristics on HSCT outcomes was not a statistically significant predictor for NRM and relapse, possibly due to the small sample size.

**Table 5:** Univariate analysis of the effect of baseline clinical characteristics on haploidentical HSCT outcomes in this cohort (*n* = 54)

Baseline factor	OS			Relapse			NRM		
	HR	95% CI	P	HR	95% CI	P	HR	95% CI	P
Male	0.93	0.71–1.29	0.626	0.96	0.6–1.52	0.752	1.05	0.64–1.46	0.834
Gender match									
Female donor to male recipient	0.89	0.59–1.23	0.496	0.98	0.39–1.59	0.628	0.88	0.59–1.63	0.658
Other	0.68	0.42–1.06	0.562	0.91	0.36–1.43	0.548	0.79	0.59–1.58	0.511
HCT-CI	1.26	1.16–1.34	< 0.02*	1.16	0.85–1.29	0.924	0.81	0.52–1.38	0.533
Disease risk index									
Low or intermediate	0.91	0.62–1.32	0.675	0.89	0.42–1.45	0.565	0.92	0.63–1.58	0.876
High or very high	1.86	1.38–2.49	< 0.04*	1.23	0.86 -1.91	< 0.56	1.01	0.76–1.64	0.268
Recipient age	0.81	0.57–1.27	0.745	0.91	0.56–1.74	0.798	0.86	0.63–1.47	0.786
Donor age	1.13	0.98–1.13	0.59	0.84	0.76–0.91	0.49	1.09	0.99–1.36	0.368
Donor- recipient -CMV status									
+ / +	1.36	0.71–1.98	0.561	1.35	0.52–2.59	0.831	1.45	0.92–1.96	0.398
-/ +	1.23	0.51–2.94	0.731	1.35	0.29–3.26	0.621	0.89	0.34–2.69	0.687
±	1.46	1.12–2.32	0.52	1.68	0.59–3.11	0.534	1.59	0.49–3.35	0.365

*OS* overall survival, *HCT-CI* Hematopoietic cell transplant-comorbidity index, *CMV* cytomegalovirus

## Discussion

Patients lacking suitable donors have found recent years' worth of success using haploidentical HSCT [3

However, while PTCy-based haploidentical HSCT can increase utilization of haplo donors in the patient's family, disease recurrence is still a major issue in the field of haplo-HSCT.

NK cell alloreactivity prediction could be regarded as a determining factor for the patient outcome and donor selection. Considering different potential haplo-donors among patient's first and second relative who share one haplotype, a data-driven instruction to prioritize haplo-donors is inevitable.

We were motivated to look into the effects of donor KIR genotypes and KIR/HLA mismatch on transplantation outcome in this research. Our results show the importance of both KIR B/x haplotype with 2DS2/2DS1 and KIR/HLA mismatch-induced NK cell alloreactivity in selecting the ideal donor for haplo-HSCT.

However, multiple investigations have shown that in the context of PTCy- or ATG-based haplo-HSCT, KIR/HLA discrepancies do not have a post-transplantation effect [39–42]. In this paper, several models for NK cell alloreactivity were tested, among them only two were significantly correlated to the outcome of the patients, including “KIR/HLA mismatch” and KIR B/x haplotype group. The comparison revealed that in the first model, the constructed KIR/HLA mismatch (such as KIR2DL1/C2, KIR2DL2,3/C1, and KIR3DL1/Bw4 mismatches) led to NK cell alloreactivity, and is correlated with better clinical outcome and improved survivals (by approximately 20% in comparison with patients in KIR/HLA match group). Our survey in this model suggests a 35% improvement in OS in the activating KIR/HLA mismatch group in comparison with KIR/HLA match group. In this paper, an interesting point to note is that the survival rate of patients in the inhibitory group KIR/HLA mismatch is perceived as preferable to the match group.

In the first model, the constructed KIR/HLA mismatch, such as KIR2DL1/C2, KIR2DL2,3/C1, and KIR3DL1/Bw4 mismatches resulted in NK cell alloreactivity, improved survivals (OS), and were correlated with better clinical outcomes.

Concerning the second model, the KIRB/x haplotype, we found favorable outcomes when the donors express activating KIR2DS2 and KIR2DS1 receptors. Our results indicated that transplants from such donors lead to decreased NRM and relapse rates and significant survival benefits. In this study, in the analyses of haplotypes, donors with haplotype A/A have a poorer OS and more NRM than those with KIR B/x-2DS1- and KIR B/x-2DS2- haplotypes. This result indicates the superiority of the expression of KIR B/x haplotypes in the overall survival rate.

Cooley et al. [21] first noted the significance of the KIR B/x haplotype, and showed that transplantation utilizing KIR B/x genotype unrelated donors was linked with markedly increased relapse-free survival in AML patients. In a follow-up study on a large cohort of acute leukemia patients who received transplants from unrelated donors (*n* = 1409), the effects of donor KIR genes were examined on the outcomes of the patients. Only two genes, KIR2DS2 and KIR2DL2, which are linked in linkage disequilibrium and located on the centromeric side of the gene cluster ("cen-B"), were correlated with less AML relapse [22]. Our findings also show that in addition to donors with KIR/HLA match structure, the KIR B/x haplotype, in the absence of KIR2DS2 and KIR2DS1, is associated with more AML relapse. Moreover, Bao et al. studied 210 patients who had hematopoietic stem cell transplants from unrelated donors and showed that selecting KIR B/x haplotype donors allowed recipients to benefit clinically more [43]. There was no noticeable difference between patients who had myeloablative unmanipulated haploidentical HSCT and those who did not have NK alloreactivity, according to study results by Russo et al. [44]. As a result of the inconsistent KIR/HLA genotyping studies in transplant centers and different communities, we looked into the effects of donor characteristics on transplant outcomes, including the role of donor KIR genotype and HLA-C1/C2 genotype, as well as the donor-recipient relationship in roughly Iranian society. Given that multiple first-degree (child, sibling, parent), second-degree (grandchild, half-sibling, etc.), or third-degree (grandparent, half-sibling) relatives may share one haplotype and possibly be haplo-donors, guidance based on these data is necessary to assist transplant centers in prioritizing haplo-donors. One of the major findings of our research was that recipient-donor pairs with KIR/HLA mismatch genetic structure had a higher frequency of acute GVHD after transplantation and a lower risk of disease relapse/progression. However, since day 100 after transplantation, during 24 months follow-up, the prevalence of chronic GVHD in the recipient-donor group with KIR/HLA mismatch decreased by about 30%, which shows the improvement of the conditions of haplo-donors with mismatch structure compared to a KIR/HLA match group.

As a result, given that Russo et al. found that many recipients with KIR/HLA incompatibility had GvHD at day 30, it may be inferred that the PTCy therapy specifically eliminates responsive alloreactive KIR2DL NK cells [44]. A more differentiated status of NK cells in transplanted individuals who developed GvHD has been demonstrated to be linked with greater blood levels of IL-15 early after HSCT [44]. The bulk of mature alloreactive NK cells is thought to be destroyed by PTCy. This activation appears to underscore a strong anti-leukemic response. Willem et al. showed that in recipients undergoing a haplo-HSCT with PTCy, NKp46 + 2B4 + NK subset evolution is faster in recipients developing GVHD or those with KIR-HLA mismatch than in those patients with relapse occurrence. The emergence of this NK cell subset early in immune reconstitution may be consistent with the beneficial impacts of GvL. Functionally, the patients developing GvHD may reflect increased granulation in vivo after NK cell activation by cellular enhancers with a high GvHD background [45].

Thus, further studies are needed to clarify the nature of cellular effectors involved in the anti-leukemia response.

Moreover, in contrast to aGvHD, the positive impact of KIR/HLA mismatch (especially activating KIR/HLA mismatch) in lowering relapse rate was a defining characteristic in our cohort. Extensive tissue damage, exposure to novel antigens, and antigenic changes associated with myeloablative conditioning (MAC) may all operate as powerful triggers of the immune responses specific to aGvHD [46]. In other words, the multiplication of recipient T cells seen after MAC may function as a catalyst for the positive effects of donor NK cells on the GvL impact and lowering the relapse rate [47]. The detection of HLA-C2 group alleles on recipient cells by KIR2DS1 on donor NK cells may cause the GvL effect or immunomodulation during allo-HSCT therapy, affecting the course of the illness. Furthermore, recipients with HLA-C2 group alleles and donors with KIR2DS1 had relapse rates that were lower than those of donors without KIR2DS1. Furthermore, we saw a change in the effect on relapse rate and NRM in the presence of activating KIR and its ligand, indicating a positive effect of KIR/HLA mismatch [48]. Other donor characteristics associated with better outcomes were donor CMV seropositivity status and kinship between donor and recipient, in addition to donor KIR genotype and KIR/HLA mismatch. CMV reactivation and illness have a significant influence on morbidity and death following transplantation despite CMV screening and antiviral treatment [49–51]. Given the rarity of CMV disease, it is believed that the rise in NRM following transplantation is most likely the result of CMV's indirect effects on immune status. Specifically, it is thought that after CMV reactivation, the expansion of Granzyme B high/CD28low/CD57high/CD8 + effector memory T cells (Tem) increases by more than 50 times, which causes a contraction of all naive T cells, including CD31 + /CD4 + putative. The integrity and heterogeneity of the remaining T-cell repertoire are negatively impacted by CMV reactivation in addition to boosting the growth of CMV-specific T-cell clones (CD8 Tem) [52, 53]. Although the significance of the donor's CMV serologic status is unknown, using a CMV- seropositive donor for a CMV- seropositive recipient is tolerated since there is a lower chance of CMV reactivation, CMV illness, and NRM [54–57]. Our findings point to a poor prognosis for CMV-seropositive recipients of organ transplants from seronegative donors (D-/R +), who were more likely to need frequent and prolonged antiviral treatment [57]. Our results indicate that sibling > 16-year donors were associated with better outcomes compared with parents who resulted in a higher incidence of NRM and higher relapse rate. Also, in younger sibling donors under 16 years compared to those over 16 years, the OS rate decreased with a slight difference. In this regard, the rise of NRM has not been significant. In this case, the possible effect of clinical variables (such as CMV reactivation and cell dose) needs to be raised and investigated in future studies.

In a study comprised of 269 AML or CML patients donated by 1 or 2 HLA-A, -B, -DR antigen-mismatched sibling or parental higher NRM and poorer outcomes were observed in the patients engrafted from their parents compared to those engrafted from their sibling [56].

The results obtained from this research indicated that KIR/HLA mismatch produced better clinical outcomes and lower relapse rates for patients receiving PTCy-based haploidentical PBSC transplantation.

## Conclusion

NK cell alloreactivity prediction based on KIR/HLA mismatches, as well as donor selection based on KIR genotypes may be useful to maximize HSCT results owing to the increased number of prospective haplo-donors and due to donors-recipients HLA incompatibility. Furthermore, we found donor risk characteristics that make it possible for a haplo-HSCT donor selection algorithm to be more efficient. Our study's tiny sample size, however, is its principal drawback compared with studies in related donor transplantation assessing HLA disparity, KIR genotyping, and other clinical factors, which often examine hundreds of patients. Considering the limitation of financial resources, time constraints, the limited sample size, and missing data due to difficulties in patient monitoring, future research is needed to confirm these findings by replicating results in a large number of contributors. Also, in our series, all patients received peripheral blood stem cells as the stem cell source, and thus the ability to extrapolate these results to haplo-marrow transplantation with PTCy is less clear.

## Methods

### Patient characteristics

Fifty four AML patients who had their first T cell-replete haploidentical hematopoietic stem cell transplant between 2018 and 2021 were included in the study by the Research Institute for Oncology, Hematology, and Cell Therapy (RIOHCT), Tehran, Iran. All participants provided informed consent for the study, which was approved by the Tehran University of Medical Sciences (TUMS) Committee for Medical Ethics [IR.MUI.REC.1400.036]. The demographic and clinical characteristics of the patients are listed in Table 1. Only patients who received a transplant of T cell-replete peripheral blood hematopoietic stem cells were included in the study.

### HSCT parameters, condtioning regime and GvHD Prophylaxis

All patients and their donors underwent high-resolution HLA molecular typing for the HLA-A, -B, -C, -DRB1, and -DQB1 loci. This was done using a high-resolution Sanger sequence-based technique. Because HLA donor-specific antibodies (DSAs) may induce primary graft failure in HLA-mismatched allografts, DSAs screening was conducted on all recipients. If a pre-HSCT bone marrow test revealed morphologically full remission, HSCT was carried out on the patient despite the existence of minimally refractory disease (CR).

**Fig. 4:**
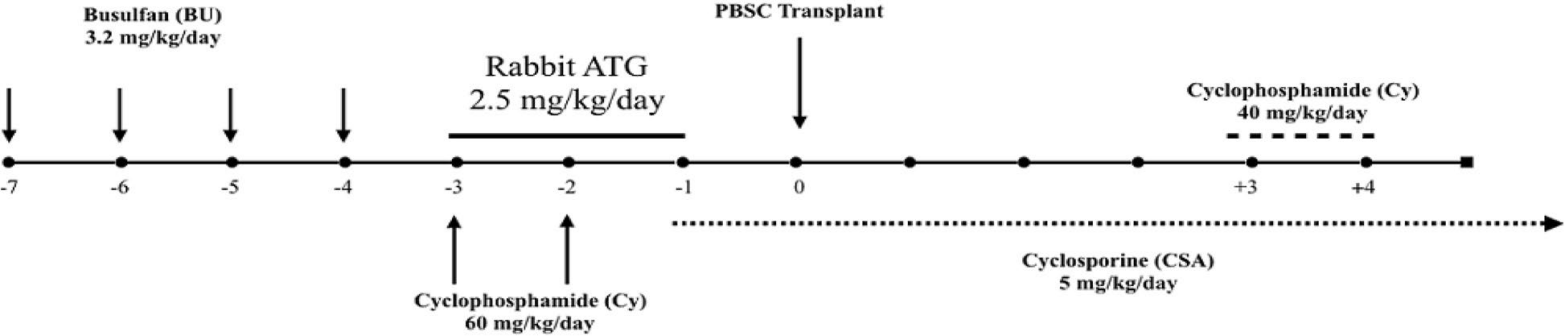
conditioning regimens, conditioning intensity, GvHD prophylaxis used, PTCY dose and timing as well as use of ATG

Patients received irradiation-free myeloablative conditioning (MAC) regimen including busulfan (a total dose of 3.2- 4.8 mg/kg/day, according to patients' ideal body weight, from day -6 to -3), cyclophosphamide (60 mg/kg/day, on days -2 and -1) plus rabbit anti-human thymocytes globulins (ATG-Thymoglobuline, Sanofi, 2.5 mg/kg/day from days -3 to -1). T cell replete PBSC grafts were infused on day 0. The GvHD prophylaxis was based on administration of cyclosporine A (CsA) started from day -1 and also PTCy (cyclophosphamide 40 mg/kg/day i.v.) on days + 3 and + 4 (Fig. 4). Patients started receiving Granulocyte Colony-Stimulating Factor (G-CSF) on day + 5.Based on their serological status, patients were divided into low-risk (donor [D]-/recipient [R]-], intermediate-risk (D^+^/R^−^) or (D^+^/R^+^), and high-risk groups (D-/R +) in order to account for the risks of post-transplant cytomegalovirus (CMV) reactivation [30]. KIR genotyping and KIR/HLA Matching of Donor–Recipient Pairs.

All donors were screened for the presence, or lack of KIR genes, including inhibitory (KIR2DL1-3, 2DL5A/B, 3DL1-3) and activating (KIR2DS1-5, 3DS1) genes, and KIR genotypes were divided into A and B haplotypes based on gene content. KIR A/A is homozygous for group A haplotypes, while KIR B/x haplotypes were defined as the presence of several activating KIR genes and at least one of the KIR2DL2, KIR2DL5, KIR2DS1, KIR2DS2, KIR2DS3, and KIR2DS5 genes [31]. An inhibitory KIR/HLA mismatch was shown by the presence of the KIR genes encoding KIR2DL1, KIR2DL2, and KIR3DL1 in the donor and the absence of the corresponding homologous Ligands in the recipient. The inhibitory KIR/HLA mismatches identified in this research are KIR2DL1/C2, KIR2DL2,3/C1, and KIR3DL1/Bw4 [32, 33]. Another sign of an activating KIR/HLA mismatch was the existence of KIR genes encoding KIR2DS1 and KIR2DS2 in the donor and the homologous Ligands in the recipient.

### Definitions and study endpoints

Overall survival (OS) is the likelihood of surviving, regardless of the stage of the illness at any given moment. The likelihood of dying without experiencing a relapse after HSCT is known as non-relapse mortality (NRM). The likelihood of experiencing a disease recurrence is known as the relapse incidence (RI). On days + 15, + 30, + 60, and + 90 after HSCT, donor chimerism was assessed. If clinically necessary, it was also assessed in whole bone marrow mononuclear cells by quantitative PCR of instructive short tandem repeats in the donor and recipient. > 0.5 × 109/L neutrophils and > 20 × 109/L platelets for three days in a row without blood transfusion assistance were considered sustained donor cell engraftment. Regardless of peripheral cell blood levels, graft rejection was defined as a lack of initial engraftment of donor cells (primary graft failure) or a loss of donor cell engraftment (secondary graft failure). According to the stated criteria, acute GvHD (aGvHD) and chronic GvHD (cGvHD) diagnoses and grades were made.

## Statistical Methods

The statistical program GraphPad Prism v8.0 was used for all calculations (San Diego, CA). Meier-Kaplan analysis was used to compare the survival (OS) and relapse (RR) rates of the patients based on a variety of factors, including KIR-HLA inc, donor age, relationship, CMV, 2DS1, 2, and RC2 + , and the survival curve of the patients was compared using the Long-Rank test at the confidence level of 0.95 (P-value = 0.05). The relevance of KIR/HLA mismatches contributing to the incidence of aGVHD and cGVHD was examined using the chi-squared test. The effects of baseline clinical factors on survival outcomes were analyzed using univariate models, and the effects of these factors on OS, PFS, relapse incidence, and NRM were determined by univariate Cox regression analyses.

Baseline clinical factors included in the univariate models were recipient age, sex, recipient-donor sex combination (female donor to male recipient vs. others), hematopoietic cell transplant comorbidity index (HCT-CI), donor age (continuous), recipient-donor CMV serostatus.

## Data Availability

The raw data supporting the conclusions of this article will be made available by the authors, without undue reservation. For more information, please contact to “ahmadvand.mohamad64@yahoo.com”.

## Abbreviations

AML: Acute myeloid leukemia
CMV: Cytomegalovirus
GvL: Graft versus leukemia
GVHD: Graft-versus-host disease
HSCT: Hematopoietic stem cell transplantation
NRM: Non-relapse mortality
OS: Overall survival
PTCy: Post-transplant cyclophosphamide
TRM: Transplant-related mortality
